# Long term follow up for children with congenital heart disease. For want of a nail the Kingdom was lost

**DOI:** 10.1038/s41390-025-04032-x

**Published:** 2025-04-05

**Authors:** Nadia Badawi, Natalie Fairbairn, Himanshu Popat

**Affiliations:** 1 CP Alliance Professor of Cerebral Palsy, Cerebral Palsy Alliance Research Institute, The University of Sydney, Medical Director, Co-Head Grace Centre for Newborn Intensive Care, The Children’s Hospital at Westmead, Corner Hawkesbury Road and Hainsworth Street, Locked Bag 4001, WESTMEAD NSW 2145, Sydney, Australia; 2 Occupational Therapist and Research Officer, Grace Centre for Newborn Intensive Care, The Children’s Hospital at Westmead, Corner Hawkesbury Road and Hainsworth Street, Locked Bag 4001, WESTMEAD NSW 2145, Sydney, Australia; 3 Clinical Lead, Co-Head of the Department, Grace Centre for Newborn Intensive Care, The Children’s Hospital at Westmead, NHMRC CTC Future Leader Fellow, NHMRC Clinical Trial Centre, Camperdown, The University of Sydney, Chair, Neonatal Intensive Care Unit Study group, NSW, Sydney, Australia

Australia has an excellent healthcare system which is considered to be one of the best in the world, providing safe, affordable and high-quality care. It ranks first among OECD countries for healthcare outcomes including effective treatments, recovery and equity, and third overall for healthcare performance.^1^ Australians have access to specialized neonatal care and a relatively low neonatal mortality rate compared with many other countries. Despite this there are areas for improvement, particularly in access to coordinated care following discharge from hospital. This is exemplified among children with congenital heart disease (CHD) who are increasingly surviving to adulthood, of whom some will go on to have their own children with congenital heart disease.^2^

Children with CHD are at a higher risk of neurodevelopmental, behavioural and psychological problems as well as cerebral palsy and developmental coordination disorder.^3^ Regular and systematic follow up by a trained multidisciplinary team including pediatric cardiologists, developmental pediatricians, psychologists and allied health professionals enables the early detection of problems or delays. Early identification of delay allows for early intervention to maximize the child’s development, academic performance, social participation and well-being. This can significantly improve long term outcomes for the children and their families, not to mention the health economic benefits. This multidisciplinary follow up should include cognitive, social, neurological and psychological surveillance and support which is tailored to the child’s and family’s specific needs.

Families require support to help them understand the consequences of their child’s condition, as well as any associated conditions such as chromosomal abnormalities, syndromes or other birth defects, to help them manage expectations, and to access resources and support networks. These support systems should persist into adulthood in order to provide these children and their families with comprehensive surveillance and support across the lifespan, ensuring that they can reach their full potential despite the challenges posed by their heart condition.^4^

The current paper by Abell et al. is a study that highlights how current neurodevelopmental follow-up care for children with CHD in Australia is largely unfunded, fragmented, inconsistent, uncoordinated and does not meet the needs of children and their families. In Australia, follow up is integrated into existing healthcare services rather than being centralized as in some North American cardiac programs. This can be beneficial for accessibility but might lack the specialized focus some patients need. They suggest an approach which would leverage common pathways and utilize community-based primary care services, pediatric clinics, child development services, neonatal follow-up programs, and allied health providers which would allow more flexible and accessible care tailored to local contexts and needs. The authors also discuss the significant variation in availability and access to these services across different regions which leads to disparities in care quality and outcomes. It is widely recognized that people living in regional and rural areas, which is a particular issue for Australia with its widely dispersed population, have worse health outcomes, and this inequity is exaggerated in people with uncommon conditions.

Systematic neurodevelopmental follow up programs throughout Australia are needed in line with the recent Australian National Standards of Care for Children with CHD. These guidelines recognize the need for embedding neurodevelopmental surveillance as standard of care for all children with complex CHD.^5^ Following the release of these National Standards, the authors designed the current qualitative interview-based study to assess service delivery of care supporting neurodevelopmental needs of children with CHD in Australia. While the study had under a 50% response rate, this may be less important with a qualitative study, and the results reflect our own experiences as clinicians. Of particular note was the finding that coordination of follow up was often left to the family to manage and that infants received follow up only if problems were detected. Furthermore, when pediatricians were doing the follow up, the tests and schedule depended on the pediatrician’s experience with particular assessments. These findings explain much of the inconsistency in developmental follow up for children with CHD.

Neonatal Intensive Care Units (NICUs) in Australia and New Zealand have comprehensive and well-established developmental follow-up programs to monitor and support the growth and development of high-risk newborns, however most developmental follow up programs do not offer neurodevelopmental follow up for infants who have surgical needs (unless they are born extremely pre-term or of low birth weight). Despite the plethora of evidence of the increased risks of neurodevelopmental delay and the need for systematic surveillance for infants who undergo major surgery both cardiac and non-cardiac in the newborn period, their needs have largely been ignored. As newborn cardiac surgery is very expensive, often costing hundreds of thousands of dollars depending on the complexity of the procedure, it is inconceivable that neurodevelopmental follow-up, which is much less costly but can significantly improve long-term outcomes for children with CHD is so underfunded.

The Australian and New Zealand Neonatal Network plays a pivotal role in collaborating with NICUs across both countries to collect data, improve clinical practices and produce annual reports which describe the survival and developmental outcomes of these infants.^6^ As infants with CHD survive increasingly complex procedures that are performed at earlier gestations and smaller birthweights they are receiving more attention with the recent establishment of the neonatal surgery network.^7^

The authors of this current national survey of practice in Australia have successfully highlighted the disjointed, inconsistent and uncoordinated follow up of children who have undergone surgery for CHD. The National Standards have had significant input from health professionals, people with CHD and their families and are a blueprint for neurodevelopmental and psychological follow up to facilitate timely, appropriate and evidenced-based assessment and intervention. Developing adequately funded, equitable, accessible and multidisciplinary surveillance programs for children with CHD in Australia is a vital component of their ongoing care. The current study has identified the significant weaknesses of current neurodevelopmental follow-up care in Australia which is at odds with a health system which is excellent in so many other ways.
